# Menthol Binding and Inhibition of α7-Nicotinic Acetylcholine Receptors

**DOI:** 10.1371/journal.pone.0067674

**Published:** 2013-07-23

**Authors:** Abrar Ashoor, Jacob C. Nordman, Daniel Veltri, Keun-Hang Susan Yang, Lina Al Kury, Yaroslav Shuba, Mohamed Mahgoub, Frank C. Howarth, Bassem Sadek, Amarda Shehu, Nadine Kabbani, Murat Oz

**Affiliations:** 1 Departments of Pharmacology Laboratory of Functional Lipidomics, College of Medicine and Health Sciences, UAE University, Al Ain, United Arab Emirates; 2 Department of Molecular Neuroscience, George Mason University, Fairfax, Virginia, United States of America; 3 School of Systems Biology, George Mason University, Fairfax, Virginia, United States of America; 4 Department of Biological Sciences, Schmid College of Science and Technology, Chapman University, Orange, California, United States of America; 5 Department of Physiology, College of Medicine and Health Sciences, UAE University, Al Ain, United Arab Emirates; 6 Department of Computer Science, George Mason University, Fairfax, Virginia, United States of America; Centre for Addiction and Mental Health, Canada

## Abstract

Menthol is a common compound in pharmaceutical and commercial products and a popular additive to cigarettes. The molecular targets of menthol remain poorly defined. In this study we show an effect of menthol on the α_7_ subunit of the nicotinic acetylcholine (nACh) receptor function. Using a two-electrode voltage-clamp technique, menthol was found to reversibly inhibit α7-nACh receptors heterologously expressed in *Xenopus* oocytes. Inhibition by menthol was not dependent on the membrane potential and did not involve endogenous Ca^2+^-dependent Cl^−^ channels, since menthol inhibition remained unchanged by intracellular injection of the Ca^2+^ chelator BAPTA and perfusion with Ca^2+^-free bathing solution containing Ba^2+^. Furthermore, increasing ACh concentrations did not reverse menthol inhibition and the specific binding of [^125^I] α-bungarotoxin was not attenuated by menthol. Studies of α_7_- nACh receptors endogenously expressed in neural cells demonstrate that menthol attenuates α_7_ mediated Ca^2+^ transients in the cell body and neurite. In conclusion, our results suggest that menthol inhibits α7-nACh receptors in a noncompetitive manner.

## Introduction

Menthol is a monocyclic terpene alcohol used widely as a flavoring and cooling additive in a number of pharmaceutical and commercial products [1], [2]. It is used by the tobacco industry to mask the harshness, increase the ease of smoking and provide a cooling sensation that appeals to many smokers [3]. In fact, menthol has been reported to be present in varying concentrations in 90 percent of tobacco products [4]. Menthol as an additive has come under close scrutiny following recent FDA reports [5] suggesting that it may facilitate smoking behavior and promote an adverse effect of smoking on health. Evidence also suggests that smoking of mentholated cigarettes is more prevalent in racial/ethnic minority populations and that smokers of mentholated cigarettes tend to smoke fewer cigarettes per day than regular cigarette smokers (for reviews, [6], [7], [4]). An association between smoking menthol cigarettes and a greater difficulty in quitting smoking is also greater in racial/ethnic minority populations as well as young smokers [4].

Nicotine, an alkaloid found in the tobacco, is considered to mediate most of the pharmacological and addictive properties of tobacco via its direct actions on nicotinic acetylcholine (nACh) receptors (for a review, [8]). Interaction between menthol and nACh receptors has been examined previously both *in vivo* and *in vitro* [9], [10], [11], [12]. For example, irritation and sensory perception induced by nicotine [9] and cigarette smoke inhalation [11] are significantly reduced by menthol. In addition to sensory responses, nicotine-induced decreases in body temperature, due to cutaneous vasodilation, are diminished significantly after both chronic and acute menthol administrations [10]. Menthol's ability to trigger the cold-sensitive transient receptor potential melastatin (TRPM) receptor is thought to be a mechanism for the cooling sensation it provokes when inhaled, eaten, or applied to the skin.

In the central nervous system, the nACh receptor can be broadly divided into two classes, heteromeric β-subunit containing receptors and homomeric α7-type receptors [13], [14]. Recently menthol has been shown to regulate the function [12] and expression [15] of α4β2-nACh receptors in the brain. To date however, little is known about menthol actions on other nACh receptors. In this study, we have tested the hypothesis that menthol modulates the function of the calcium conducting α7-nACh receptor. We have examined the effects of menthol on the function of human α7-nACh receptors expressed in *Xenopus* oocytes and rat α7-nACh receptors endogenously expressed in cultured neural cells. Our findings reveal a novel role for menthol in the modulation of α7-nACh receptors and suggest that this compound may contribute to cholinergic transmission as well as nicotine addiction.

## Materials and Methods

### Recordings from oocytes

Mature female *Xenopus laevis* frogs were purchased from Xenopus Express (Haute-Loire, France), housed in dechlorinated tap water at 19–21°C with a 12/12-hour light/dark cycle, and fed food pellets supplied by Xenopus Express. The procedures followed in this study were in accordance with the Guide for the Care and Use of Laboratory Animals (8^th^ edition) of the National Institutes of Health (Bethesda, MD) and approved by the Institutional Animal Care and Use Committee at the UAEU. Clusters of oocytes were removed surgically under benzocaine (Sigma, St. Louis, MO) local anesthesia (0.15% w/V), and individual oocytes were dissected manually in a solution containing (in mM): NaCl, 88; KCl, 1; NaHCO_3_, 2.4; MgSO_4_, 0.8; HEPES, 10 (pH 7.5). Dissected oocytes were then stored 2–7 days in modified Barth's solution (MBS) containing (in mM): NaCl, 88; KCl, 1; NaHCO_3_, 2.4; CaCl_2_, 2; MgSO_4_, 0.8; HEPES, 10 (pH 7.5), supplemented with sodium pyruvate, 2 mM, penicillin 10,000 IU/L, streptomycin, 10 mg/L, gentamicin, 50 mg/L, and theophylline, 0.5 mM. Briefly, oocytes were placed in a 0.2 ml recording chamber and superfused at a rate of 2–3 ml/min. The bathing solution consisted of (in mM): NaCl, 95; KCl, 2; CaCl_2_, 2; and HEPES 5 (pH 7.5). The cells were impaled with two glass microelectrodes filled with a 3 M KCl (1–5 MΩ). The oocytes were routinely voltage clamped at a holding potential of −70 mV using a GeneClamp-500 amplifier (Axon Instruments Inc., Burlingame, CA). During experiments on the current-voltage relationship of ACh-responses, membrane potentials from −100 to −20 mV were held for 30 sec to 1 min and then returned to −70 mV.

Drugs were applied by gravity flow via a micropipette positioned about 2 mm from the surface of the oocyte. Some of the compounds were applied externally by addition to the superfusate. All chemicals used in preparing the solutions were from Sigma-Aldrich (St. Louis, MO). Racemic, (−) and (+)-menthol, acetylcholine, and α-bungarotoxin were obtained from Sigma (St. Louis, MO). Procedures for the injections of BAPTA (50–100 nl, 100 mM) were performed as described previously [16]. BAPTA was prepared in Cs_4_-BAPTA and injections were performed 1 hr prior to recordings using an oil-driven ultra microsyringe pump (Micro4, WPI, Inc. Sarasota, FL). Stock solutions of menthol used in this study were prepared in ethanol at a concentration of 10 mM.

cDNA plasmids for human α7-nACh receptor expression were kindly provided by Dr. J. Lindstrom (University of Pennsylvania, PA). Capped cRNA transcripts were synthesized *in vitro* using a mMESSAGE mMACHINE kit from Ambion (Austin, TX) and analyzed on a 1.2% formaldehyde agarose gel to check the size and quality of the transcripts.

### Radioligand binding studies

Oocytes were injected with 10 ng human α_7_-nicotinic acetylcholine receptor cRNA, and the functional expression of the receptors was tested by electrophysiology after 2 days. Isolation of oocyte membranes was carried using a published method [17]. Briefly, oocytes (200–300 oocytes per assay) were suspended (approximately 20 µl/oocyte) in a homogenization buffer containing HEPES 10 mM, EDTA 1 mM, 0.02% NaN_3_, 50 µg/mL bacitracin, and 0.1 mM PMSF (pH 7.4) at 4°C on ice and homogenized using a motorized Teflon homogenizer (six strokes, 15 sec each at high speed). The homogenate was centrifuged for 10 min at 800× *g*. The supernatant was collected and the pellet was suspended in homogenization buffer and centrifuged at 800× *g* for 10 min. Supernatants were combined and centrifuged for 1 hr at 36000× *g*. The membrane pellet was suspended in homogenization buffer and used in the binding studies.

Binding assays were performed in 500 µL of binding buffer (in mM; NaCl, 140; KCl, 2.5; CaCl_2_, 2.5; MgCl_2_, 1; HEPES 20; pH 7.4) containing 50 µL of oocyte preparation and 0.1–5 nM [^125^I] α-bungarotoxin (2200 Ci/mmol; Perkin-Elmer, Inc. Waltham, MA). Nonspecific binding was determined using 10 µM α-bungarotoxin. Oocyte membranes were incubated with [^125^I] α-bungarotoxin in the absence and presence of drugs, for 1 hr at room temperature (22–24°C). The radioligand was separated by rapid filtration onto GF/C filters presoaked in 0.2% polyethyleneimine. Filters were then washed with two 5 ml washes of ice-cold binding buffer, and the radioactivity was determined by counting samples in a Beckman Gamma-300 γ-counter.

### [^125^I] α-bungarotoxin binding in intact oocytes

2–3 days after injection, [^125^I] α-bungarotoxin binding assays were performed subsequent to the voltage-clamp measurement on the same intact oocyte. A cellular current response (to 100 µM ACh) of more than 3000 nA was used as an inclusion criteria in the binding assay. Notably, most oocytes had maximum current amplitude of 4000 to 6000 nA. Binding assays in single intact oocytes were carried out by modification to an existing method [18]. Briefly, oocytes were incubated in 20 nM [^125^I] α-bungarotoxin, 5 mg/mL BSA, MBS at room temperature for 2 hr. Non-injected oocytes were incubated under the same conditions to measure non-specific binding. Excess toxin was removed by washing each oocyte with 25 mL of MBS. Radioactivity was measured using a Beckman Gamma-300 γ-counter. Counts per minute (cpm) values were calculated from the mean of 4 separate experiments. In each experiment, 5–6 oocytes were used per group.

### Cell Culture and Immunocytochemistry

Pheocromocytoma line 12 (PC12) cells were grown on a rat collagen (50 µg/mL, Gibco) matrix using dMEM containing 10% horse serum, 5% fetal bovine serum (FBS), and 1% penicillin-streptomycin (Pen-strep) antibiotic as previously described [19]. Cells were differentiated with the addition of 10 nM 2.5S nerve growth factor (NGF) for 2 days prior to transfection and imaging (Prince Laboratories). Cells were transfected with a pCMV-GCaMP5G plasmid (Addgene) using Lipofectamine 2000 (Life Sciences). An empty pEGFP-N1 plasmid was used as a vector control.

Cell fixation and immunocytochemistry was performed on PC12 cells as described [20]. In brief cells were fixed with 0.3% glutaraldehyde and permeabilized with 0.05% Triton X-100. Cells were stained with a fluorescently labeled AlexaFluor 647 α-bungarotoxin (fBgtx) (Life Sciences) and a rhodamine phalloidin antibody (Cell Signaling). Stained cells were visualized using a Nikon Eclipse 80i confocal microscope fitted with a Nikon C1 CCD camera. Images were captured using AxioVision and EZ-C1 software.

### Calcium Imaging

Calcium imaging was performed using the genetically encoded calcium sensor protein GCaMP5G (Addgene) (Ackerboom et al, 2012). This method was preformed essentially as described [21] with some minor modifications. Briefly, PC12 cells cultured on 8 mm coverslips were placed into a recording chamber and perfused with a recording buffer (in mm; NaCl, 110; KCl, 5.4; CaCl_2_, 1.8; MgCl_2_, 0.8; D-glucose, 10; HEPES, 10 at pH 7.4 (adjusted with NaOH)). Image exposure time was set to 100 msec and pixel binning was set to 2×2. Neutral density filters were used to reduce photobleaching. Imaging was carried out at room temperature (22°C) for 30 seconds at an acquisition rate of one image every 500 msec. Drugs were applied via a perfusion bath after 10 seconds of baseline recording. Baseline fluorescence readings were taken before drug exposure in 30 s intervals for 5 min (a total of 10 readings). For images presented here, baseline readings were shortened to five readings. For menthol and Bgtx applications, cells were preincubated with HBSS+10 mM HEPES and menthol or Bgtx for 20 min prior to calcium imaging. Regions of interest (ROIs) within the neurite and soma were chosen based on co-detection of GCaMP5 and fBgtx. Images were taken using Zeiss Observer 7.1 fitted with an AxioCam MRm camera and images were captured using the AxioVision software. Camera intensification was set to keep exposure times <50 ms for GCaMP5; pixel intensities were <25% of saturation. GCaMP5 fluorescence was acquired with a 488 nm laser and 535/30 emission filter.

A total of 40 cells per experimental group (n = 40) were used to obtain the average values. Analysis of the fluorescence was performed using ImageJ (NIH). A fluorescent signal above two standard deviations of the mean, from the baseline, was determined as an inclusion criterion in the analysis in order to dismiss random fluctuations.

### Structural Modeling

Docking of L-menthol (1R,2S,5R) to the -nACh muscle receptor was performed using the structure of L-menthol (ZINC ID: 01482164) from the ZINC Vr. 12 Database [22]. A crystal structure for the muscle nACh receptor was obtained from the Protein Data Bank [23] under PDB ID 2BG9 [24]. This receptor was chosen as it is the only complete nACh receptor available in the PDB and it shares close structural homology with the α7-nACh receptor [25]. Rigid docking simulations were performed using AutoDock 4.2 [26] and the Molecular Graphics Laboratory Tools (MGLTools) Vr. 1.5.4 rev. 30 [27], [26]. Ligand and receptor files were prepared using recommended procedures described in the MGLTools software documentation (http://mgltools.scripps.edu/documentation). Two torsion angles were specified as parameters for the ligand, while the receptor was modeled as a rigid structure. A grid box area was specified to for AutodDock to bind the ligand on relevant regions of the receptor's molecular surface. Specification of the grid box area took into account the similar binding characteristics believed to be shared by propofol and menthol [28], and the close homology of the gamma-aminobutyric acid receptor (GABA_A_R) to nACh muscle receptor [25]. The grid box was set to include key residue positions evaluated by Williams and Akabas [29] for testing propofol binding to the GABA_A_R-α_1_ segment. These key residues were mapped onto the muscle nACh and α7-nACh (UniProt AC: P36544) receptor sequences through a multiple sequence alignment, using MUSCLE Vr. 3.8.31 [30]. Once the grid box area was set to include these residues, docking simulations were performed in AutodDock through the Lamarkian Genetic Algorithm with default parameters. In order to obtain convergence, the “maximum number of evaluations” was increased to “long.” Analysis of the generated docked conformations for the ligand was performed using MGLTools. Image rendering was performed using VMD 1.9 [31].

### Data analysis

Average values were calculated as the mean ± standard error means (S.E.M.). Throughout this study, n defines the number of oocytes or number of samples tested in each experiment. Statistical significance was analyzed using Student's *t* test or ANOVA as indicated. Concentration-response curves were obtained by fitting the data to the logistic equation,
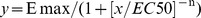
where x and y are concentration and response, respectively, E_max_ is the maximal response, EC_50_ is the half-maximal concentration, and n is the slope factor (apparent Hill coefficient).

## Results

### Menthol attenuates α7-nACh receptor activity

At the highest concentration used in this study, 1 mM acetylcholine (ACh) did not cause detectable currents in un-injected oocytes (n = 7) or in oocytes injected with distilled water (n = 6) (data not shown). Application of 100 µM ACh for 3 to 4 sec activated fast inward currents that desensitized rapidly in oocytes injected with cRNA transcribed from cDNA encoding the α_7_-subunit of human nACh receptor. Moreover, ACh-induced inward currents were abolished completely with 100 nM α-bungarotoxin (n = 7, data not shown), indicating that the α-bungarotoxin-sensitive α7-nACh receptor-ion channel mediates these responses.

**Figure 1:**
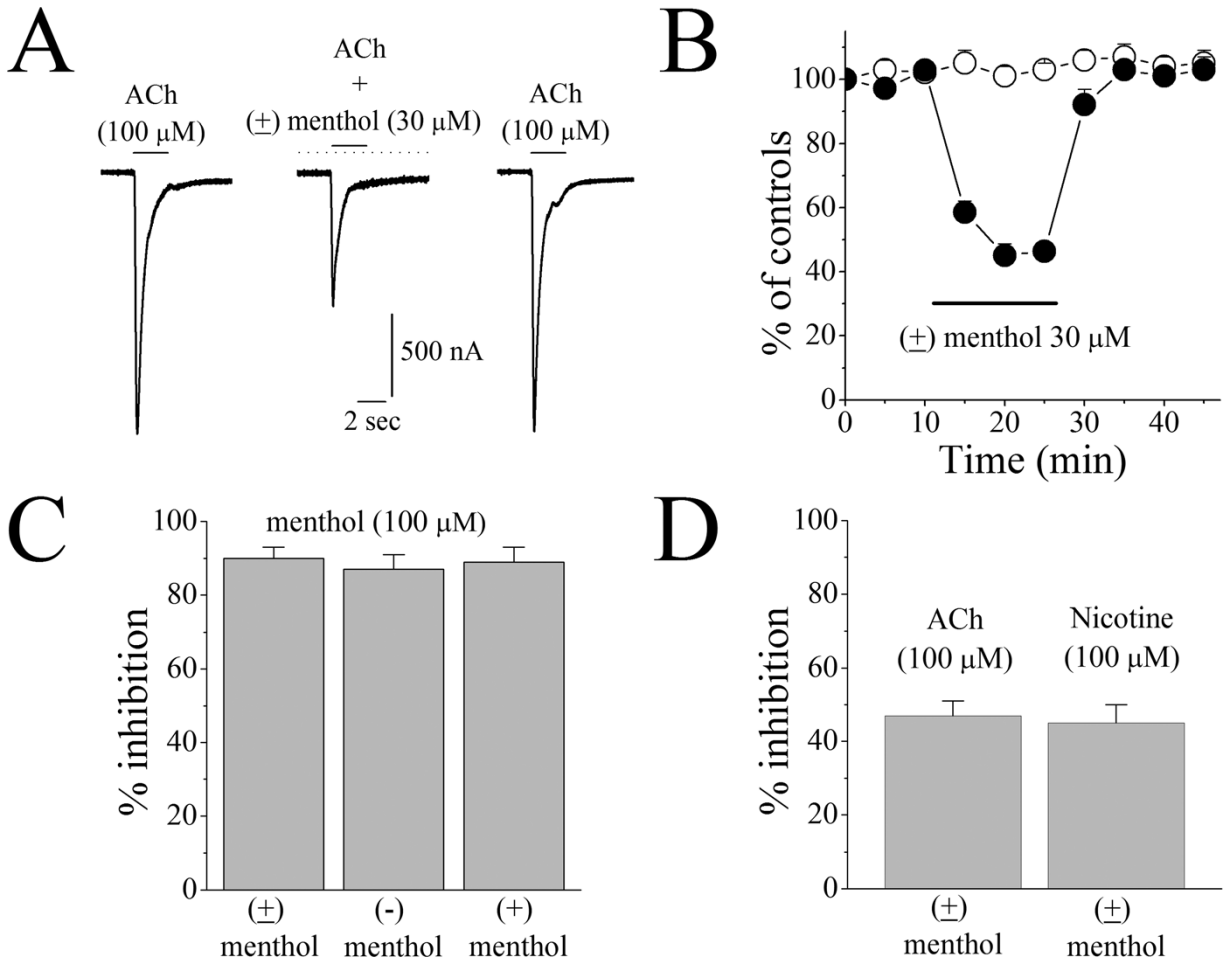
Effect of menthol on α_7_-nicotinic acetylcholine receptor-mediated ion currents. (**A**) Records of currents activated by acetylcholine (ACh, 100 µM) in control conditions (*left*), during co-application of 30 µM menthol and acetylcholine after 10 min pretreatment with 30 µM menthol (*middle*), and 15 min following menthol washout (*right*). (**B**) Time-course of the effect of menthol (100 µM) on the peaks of the acetylcholine-induced currents. Each data point represents the normalized mean ± S.E.M. of 4 to 5 oocytes. Duration of drug application is indicated by the horizontal bar in the figure. (**C**) Comparison of the extent of inhibition caused by 100 µM of (+), (−), and racemic forms of menthol application for 15 min. Bars represent the means ± S.E.M. from 6 to 7 cells. (**D**) Comparison of the effect of 30 µM of racemic menthol application for 15 min on the currents activated by 100 µM acetylcholine or 10 µM nicotine. Bars represent the means ± S.E.M. from 5 to 6 cells.

The effects of 10 min incubation with menthol (30 µM) on α_7_-nACh receptor mediated currents are shown in Fig. 1A. A time-course plot showing the effect of menthol application on the amplitudes of ACh-induced currents is presented in Fig. 1B. Whereas the vehicle solution did not alter ACh-induced currents, application of menthol (30 µM) caused a significant inhibition of the current. This inhibition by menthol was partially reversed during a washout period of 10 to 15 min. In the absence of these drugs, maximal amplitudes of currents elicited by the application of 100 µM ACh every 5 min remained unchanged during the course of the experiments (Fig. 1B, controls).

Some of the biological actions of menthol have been shown to be stereo-specific (Eccles, 1994). For this reason, we compared the effects of 100 µM of (−) and (+) stereoisomers, and racemic (±) menthol on human α7-nACh receptors. Results show that the 2 stereoisomers and the racemic menthol (100 µM) inhibit nACh receptor currents to a similar extent with no statistical significant detected between the compounds (Fig. 1C; n = 6–7, *F* (2, 16) = 0.322; ANOVA, *P*>0.05). In all subsequent experiments, unless stated, racemic (±) menthol was employed.

**Figure 2:**
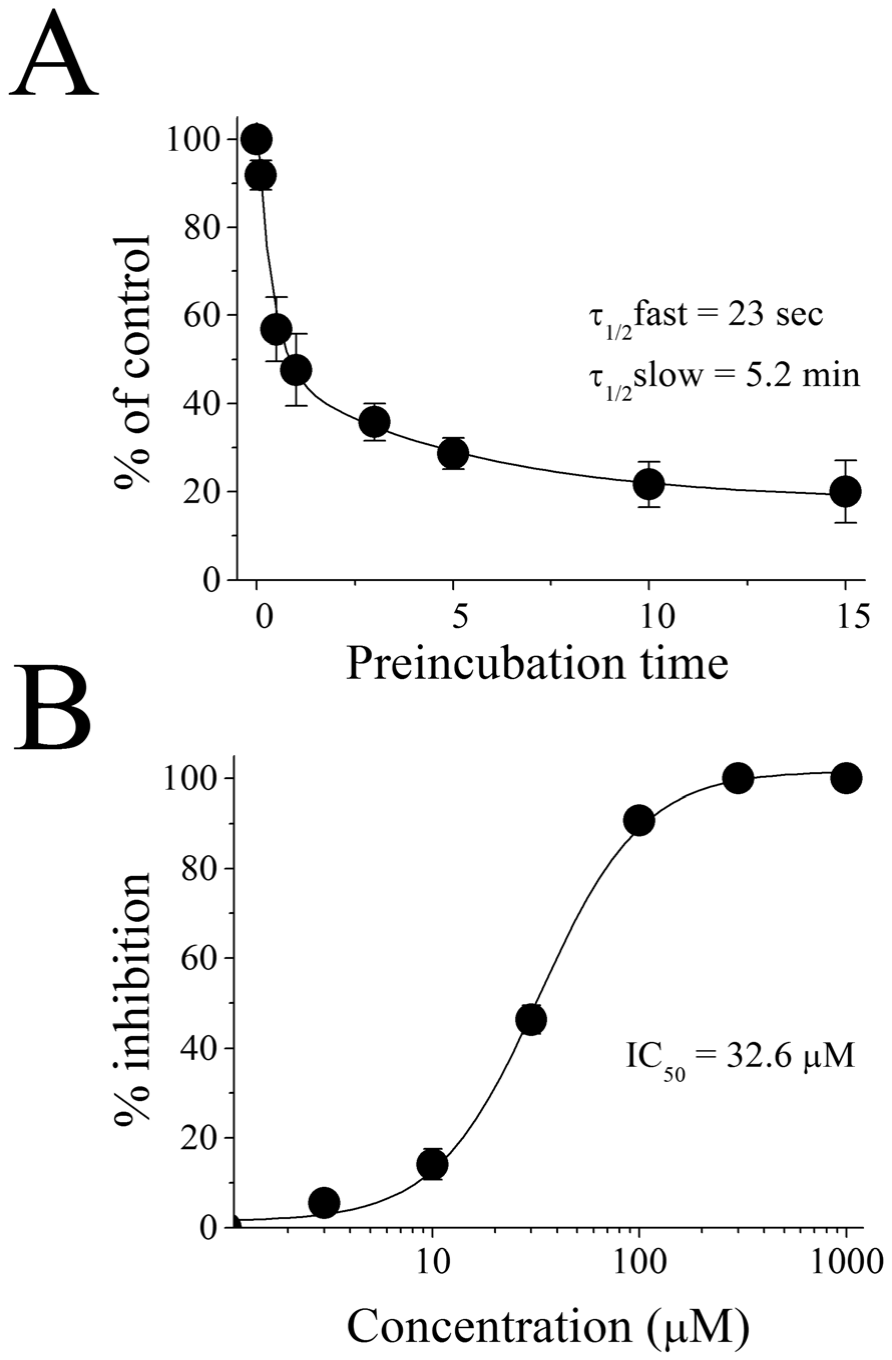
Time and concentration-dependence of menthol inhibition of α_7_-nicotinic acetylcholine receptor-mediated ion currents. (**A**) Inhibition of the α_7_-nicotinic acetylcholine receptor increases with the prolongation of menthol pre-application time. Each data point represents the mean ± S.E.M. of 5 to 6 oocytes. (**B**) Menthol inhibits α_7_-nicotinic acetylcholine receptor function in a concentration-dependent manner. Each data point represents the mean ± S.E.M. of 7 to 9 oocytes. The curve is the best fit of the data to the logistic equation described in the methods section.

Menthol is often delivered with tobacco products that contain nicotine. Therefore we tested the effect of menthol on nicotine-activated currents in oocytes. As shown in Fig. 1D, we did not find a statistically significant difference in menthol-mediated inhibition of α7-nACh receptor currents between cells treated with ACh or nicotine (n = 5–6, *F* (1, 9) = 0.052; ANOVA, *P*>0.05). It is noteworthy that the inhibitory effect of menthol was dependent on the application mode. Without menthol pre-incubation, a co-application of menthol (30 µM) and ACh (100 µM) did not alter the amplitudes of maximal currents (Fig. 2A). However after pre-incubation, menthol inhibited the maximal responses in a time-dependent manner. As incubation time was prolonged, the extent of menthol inhibition was enhanced and reached a maximum level at 10 to 15 min (Fig. 1B). Close examination of the time course of menthol actions indicated that the inhibition occurs at fast and slow phases with the respective time constants of τ_1/2fast_ = 23 sec. and τ_1/2slow_ = 5.2 min (Fig. 2A). Since the magnitude of the effect was time-dependent, menthol was applied for 10 to 15 min to ensure equilibrium conditions. Menthol inhibited the function of α7-nACh receptor in a concentration-dependent manner with respective IC_50_ and slope values of 32.6±2.3 µM and 1.7, respectively (Fig. 2B).

**Figure 3:**
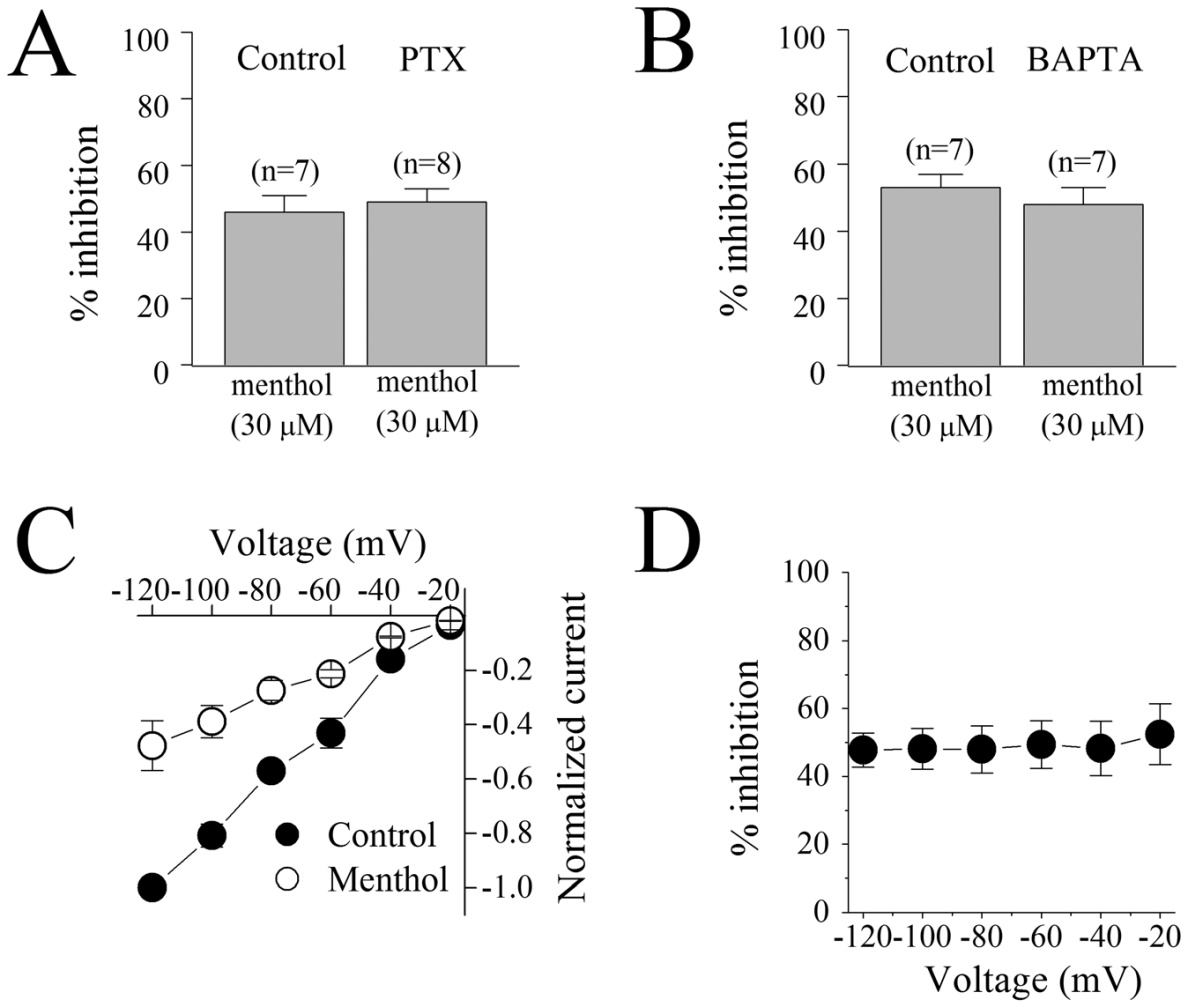
Inhibition of acetylcholine-induced currents by menthol is independent of the activation of pertussis toxin sensitive receptors, membrane potential and Ca^2+^-activated Cl^−^ channels. (**A**) Bar presentation of the effects of 30 µM menthol application (15 min) on the maximal amplitudes of ACh-induced currents in oocytes injected with 50 nl distilled-water, controls (n = 5) or 50 nl of PTX (50 µg/ml, n = 6). Bars represent the means ± S.E.M. (**B**) α_7_-nicotinic acetylcholine receptor expressing oocytes injected with 50 nl distilled water and recorded in 2 mM Ca^2+^ containing MBS solution (control) or injected with 50 nl of BAPTA (100 mM) and recorded in 2 mM Ba^2+^ containing MBS solution (BAPTA). Bars represent the means ± S.E.M. of 6 to 8 oocytes. The numbers of oocytes are presented on top of each bar. There was no statistically significant difference between menthol (30 µM) inhibition in the presence and in the absence of BAPTA injections (*P*>0.05, n = 5–8, ANOVA). (**C**) Current-voltage relationships of acetylcholine-activated currents in the absence and presence of menthol (30 µM). Normalized currents activated by 100 µM acetylcholine before (control,•) and after 15 min treatment with menthol (○). Each data point presents the normalized means and S.E.M. of five to six oocytes. (**D**) Quantitative evaluation of the effect of menthol as percent inhibition at different voltages.

G-protein coupled receptors [32] have been shown to be involved in cellular and behavioral effects of menthol. Thus, we tested the effect of menthol in control (distilled-water injected) and pertussis toxin (PTX) - injected oocytes expressing nACh receptors. There was no significant difference in menthol inhibition of ACh responses between controls and PTX-injected cells (Figure 3A, n = 7–8; *F* (1, 14) = 0.692, ANOVA, *P*>0.05 for the significance of menthol inhibition between controls and PTX group).

Since activation of α_7_-nACh receptors allows sufficient Ca^2+^ entry to activate endogenous Ca^2+^-dependent Cl^−^ channels in *Xenopus* oocytes (for a recent review: [33]), it was important to determine whether the effect of menthol was exerted on nACh receptor-mediated currents or on Cl^−^ currents induced by Ca^2+^ entry in the cell. Thus, extracellular Ca^2+^ was replaced with Ba^2+^ since Ba^2+^ can pass through α_7_-nicotinic acetylcholine receptors but causes a negligible activation of Ca^2+^-dependent Cl^−^ channels [34]. In addition to Ba^2+^ replacement, a small contribution of remaining Ca^2+^-dependent Cl^−^ channel activity has been shown to be abolished by the injection of the Ca^2+^ chelator BAPTA [34]. We tested the effect of menthol in a solution containing 2 mM Ba^2+^ in BAPTA-injected oocytes. Menthol (30 µM) produced the same level of inhibition (67±5 in controls versus 65±5 in BAPTA-injected oocytes; n = 7; *F* (1, 12) = 0.863; ANOVA, *P*<0.05) on ACh-induced currents when BAPTA-injected oocytes were recorded in Ca^2+^ free solutions containing 2 mM Ba^2+^ (Fig. 3B). Menthol has also been reported to alter intracellular Ca^2+^ homeostasis in various preparations [2]. In the oocyte expression system, an increased level of intracellular Ca^2+^ can be detected by Ca^2+^-activated Cl^−^ channels and concomitant alteration in the holding current [35], [36]. However, in control experiments, the menthol used in this study (30 µM for 15 min) did not alter the magnitudes of holding-currents in oocytes voltage-clamped at −70 mV (n = 12–14) suggesting that Ca^2+^-dependent Cl^−^ channels are not involved in the effect of menthol in our system.

Recent electrophysiological studies report that menthol inhibits the functions of Na^+^ [37], [38] and Ca^2+^ channels [38] in a voltage-dependent manner. We examined if menthol-inhibition of α7-nACh receptors was dependent on the membrane potential. As indicated in Fig. 3C, menthol (30 µM) was able to inhibit ACh (100 µM)-induced currents at all of the tested potentials and thus seemingly can act independent of voltage changes. Indeed, an evaluation of the current-voltage relationship (Fig. 3D) shows that α7-nACh receptor inhibition by menthol does not change significantly at varying holding potentials (n = 6–7, inhibition at −20 mV versus −120 mV; *F* (1, 11) = 0.058; ANOVA, *P*>0.05).

**Figure 4:**
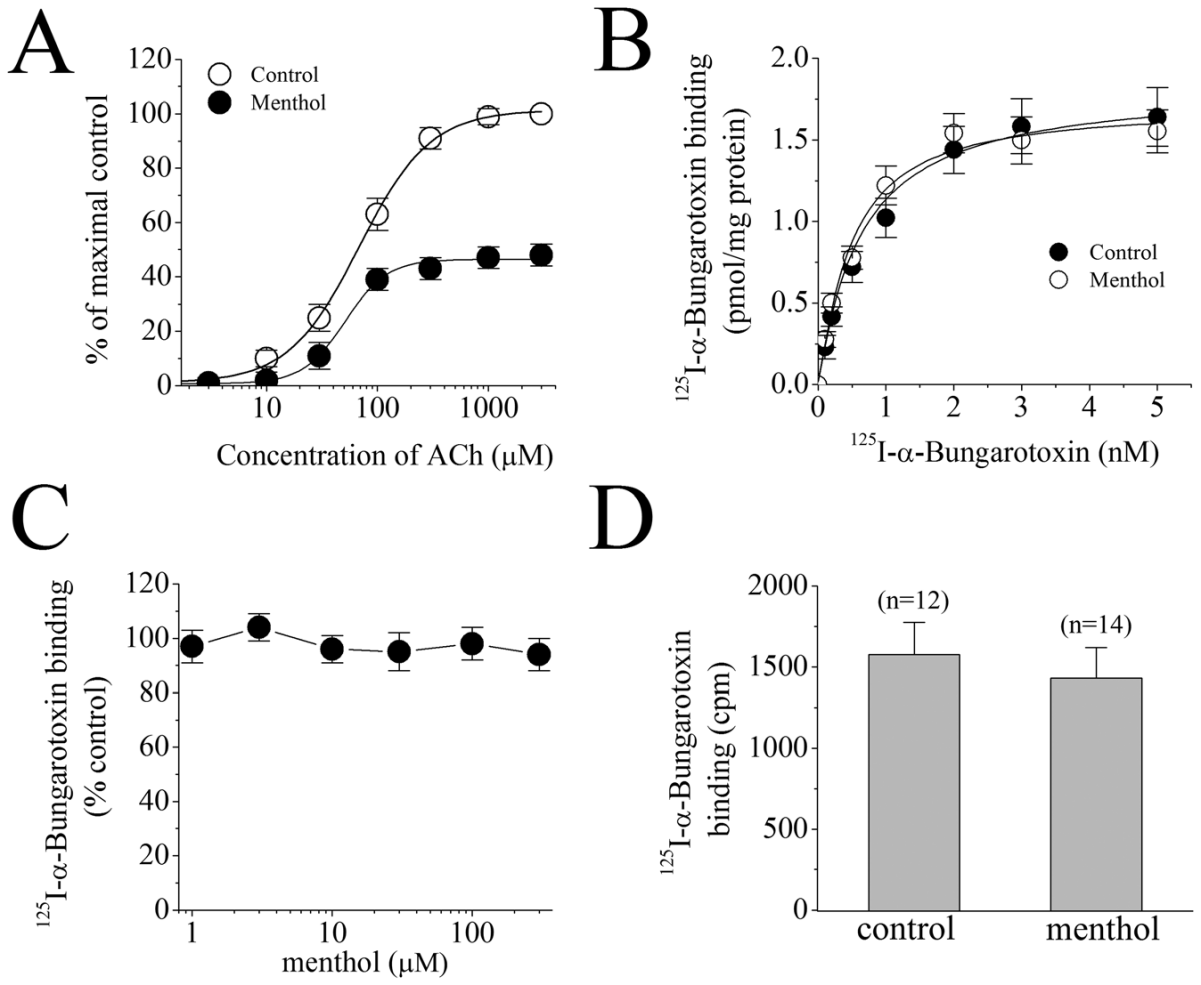
Concentration-response curves for acetylcholine-induced currents and binding of [^125^I] α-bungarotoxin in control and in the presence of menthol. (**A**) Effect of menthol on the acetylcholine concentration-response relationship. Oocytes were voltage-clamped at −70 mV and currents were activated by applying acetylcholine (1 µM to 3 mM). Oocytes were exposed to 100 µM menthol for 15 min and acetylcholine was reapplied. Paired concentration-response curves were constructed and responses normalized to maximal response under control conditions. EC_50_ and slope values were determined by fitting the curves from 6 to 8 oocytes to the standard logistic equation as described in the methods section. Data points obtained before (control) and after 15 min treatment with menthol (100 µM) were indicated by *filled circles, open circles*, and *open triangles*, respectively. Each data point presents the normalized means and S.E.M. of five to six experiments. (**B**) The effects of menthol on the specific binding of [^125^I] α-bungarotoxin to oocyte membrane preparations. In the presence and absence of menthol, specific binding as a function of the concentration of [^125^I] α-bungarotoxin is presented. Data points for controls and menthol (100 µM) are indicated by *filled circles*, and *open circles*, respectively. Data points are the means of three independent experiments carried out in triplicate.

It is possible that menthol decreases the binding of ACh to the nACh receptor by acting as a competitive antagonist at the same binding site. Concentration-response curves for ACh in the absence and presence of 30 µM menthol are presented in Fig. 4A. Menthol did not cause a significant change in the affinity of ACh for the receptor (EC_50_ values of 63±12 µM versus controls 76±11 µM; n = 6–8; *F* (1, 12) = 1.126, ANOVA, *P*>0.05), but inhibited the maximal ACh response by about 47±4% of controls (n = 6), suggesting that menthol inhibits the α7-nACh receptor response in a non-competitive manner.

We determined the effects of 30 µM menthol in radioligand binding studies using [^125^I] α-bungarotoxin. Equilibrium curves for the binding of [^125^I] α-bungarotoxin, in the presence and absence (controls) of menthol are presented in Fig. 4B. At a concentration of 30 µM, menthol did not cause a significant inhibition of the specific binding of [^125^I] α-bungarotoxin. Maximum binding activities (B_max_) of [^125^I] α-bungarotoxin were 1.9±0.3 and 1.7±0.2 pM/mg (means ± S.E.M.) for controls and menthol-treated preparations, respectively (Fig. 4B). The apparent affinity (K_D_) of the receptor for [^125^I] α-bungarotoxin was 854±236 and 716±213 pM for controls and menthol, respectively. There was no statistically significant difference between controls and menthol-treated groups with respect to K_D_ (n = 5–6, *F* (1, 9) = 1.023; ANOVA, *P*<0.05) and B_max_ K_D_ (n = 5–6, *F* (1, 9) = 1.066; ANOVA, *P*<0.05) values.

Because radioligand-binding in oocyte membrane homogenates is known to disrupt cellular integrity, the subcellular fractions used in the binding assay are likely to contain both intracellular as well as plasma membranes. To determine menthol binding and actions at the cell surface, we also performed radioligand-binding assays in intact oocytes. In these experiments, menthol (30 µM) did not cause a significant inhibition of the specific binding of [^125^I] α-bungarotoxin (20 nM) in oocytes injected with the α7-nicotinic acetylcholine receptor cRNA. Specific binding of [^125^I] α-bungarotoxin was 1576±201 cpm, 1438±189 cpm (means ± S.E.M.) for controls and menthol (30 µM)-treated oocytes, respectively. In the presence of menthol (30 µM), we did not detect a significant alteration in the specific binding of [^125^I] α-bungarotoxin in intact oocytes (n = 12–14; *F* (1, 24) = 0.026, ANOVA; *P*>0.05). Since α-bungarotoxin competes with ACh at the same binding site on the α7-nACh receptor, the current data suggests that menthol does not interact with the ACh binding site; i.e. acts as a noncompetitive antagonist.

### Menthol interacts with α7-nACh receptors in neural cells and modulates calcium signaling and neurotransmitter release

**Figure 5:**
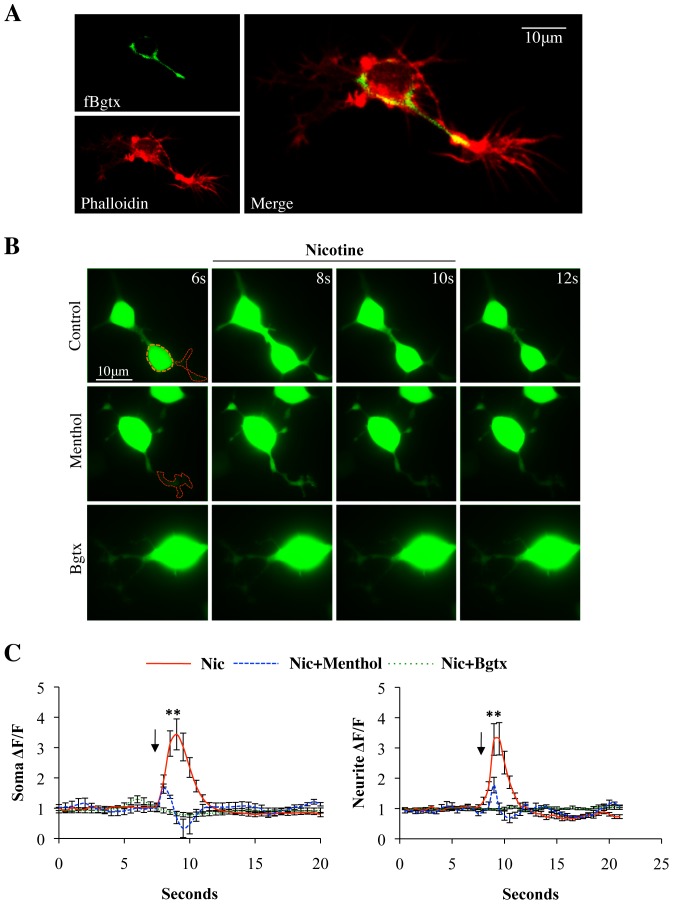
Menthol attenuates nicotine-induced calcium signaling in neural cells. **(A)** A representative PC12 cell showing the expression of α7-nACh receptors in the soma and primary neurite. Green fluorescence: fBgtx labeling; red fluorescence: anti-rhodamine phalloidin immunostaining was used to determine ROI for calcium imaging. **(B)** Live cell imaging of cells expressing the genetically encoded calcium sensor GCaMP5G. ROI within soma and neurite shown via the orange and red dotted lines, respectively. Rows top to bottom: cells pre-treated with Control (0.3% ethanol); Menthol (30μM); α-bungarotoxin (Bgtx) (50nM). Cellular images captured at 6/8/10/12 seconds (s). Nicotine (50μM) was applied at 8-10s. **(C)** Average fluorescence signal data for soma and neurite ROI imaged for 20 seconds. n = 40 cells, **P>0.01”.

**Figure 6:**
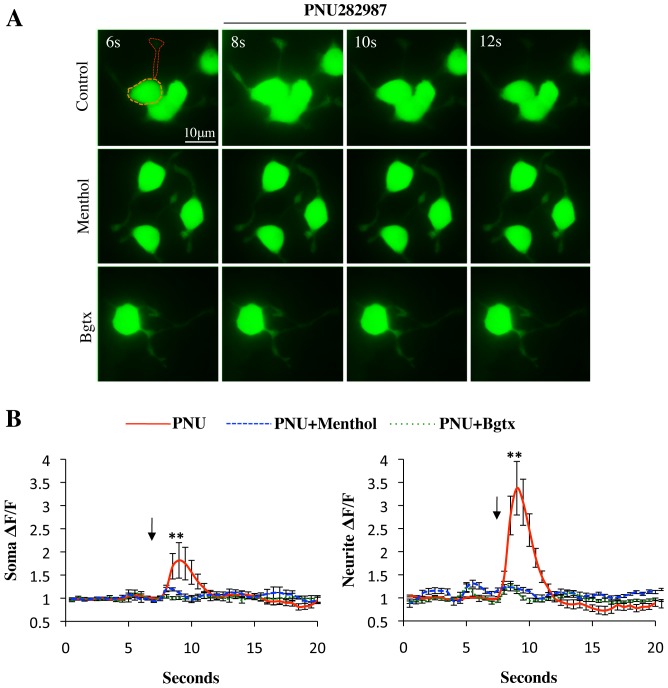
Menthol attenuates α_7_ nACh receptor calcium signaling. (**A**) Live cell imaging of cells expressing GCaMP5G. ROI within soma and neurite shown via the organge and red dotted line, respectively. ROI selection is based on co-detection of fBgtx and GCaMP5G as indicated in Fig. 5. Rows top to bottom: cells pre-treated with Control (0.3% ethanol); Menthol (30 µM); α-bungarotoxin (Bgtx) (50 nM). Image frames captured at 6/8/10/12 seconds (s). PNU (10 µM) was applied at 8–10 s. (**B**) Average fluorescence signal data for soma and neurite ROI imaged for 20 seconds. n = 40 cells, **P>0.01”.

α7-nACh receptors are endogenously expressed in PC12 cells and contribute to cellular growth and function [19]. We have utilized a culture of NGF differentiated PC12 cells to examine the effects of menthol on α_7_-nACh receptor Ca^2+^ activity in neural cells. α_7_-nACh receptors endogenous to these cells were found to be distributed in the cell body as well as neurites (Fig. 5A). Consistent with previous observation, the fluorescent α-bungarotoxin (fBgtx) signal was seen at the plasma membrane in soma and the neurites visualized with f-actin/phalloidin (Fig. 5A). α_7_-nACh receptors conduct Ca^2+^ upon activation leading to important changes in cellular signaling [14]. We validated the Ca^2+^ conducting properties of α_7_-nACh receptors in PC12 cells using the genetically encoded, high sensitivity, calcium sensor GCaMP5G [21]. Transfection of GCaMP5G into PC12 cells allowed us to assay α7-nACh receptor mediated calcium increases with and without menthol in neural cells. GCaMP5G was transiently transfected into differentiating PC12 cells 2 days prior to Ca^2+^ imaging. As shown in Figs. 5 and 6, pharmacological activation of the α_7_-nACh receptor with nicotine or the selective α_7_-agonist PNU282987 (PNU) was associated with a significant increase in intracellular Ca^2+^ within the soma and primary neurite. In particular, nicotine was found to promote a 244.3% (+/−50.8%) and 228.9% (+/−52.9%) rise in cellular Ca^2+^ levels (above basal) within the soma and neurite, respectively. PNU application was found to only mildly increase Ca^2+^ levels in the soma (81.6% (+/−38.4%)) while strongly elevating Ca^2+^ levels in the neurite (237.4% (+/−57.9%)).

We tested the effect of menthol on nicotine and PNU associated calcium changes. Cells were incubated with 30 µM menthol for 20 min prior to Ca^2+^ imaging. This pre-application of menthol was found to dramatically reduce nicotine as well as PNU mediated Ca^2+^ thus seemingly maintaining the cellular Ca^2+^ near the measured baseline (Figs. 5 and 6). In these experiments, pre-application of PC12 cells with the α_7_-nACh receptor blocker α-bungarotoxin was found to block the effects of nicotine and PNU on Ca^2+^ increase, thus confirming the specific role of α_7_-nACh receptors in the assay (Figs. 5 and 6).

### A binding site for menthol within the α7-nACh receptor

**Figure 7:**
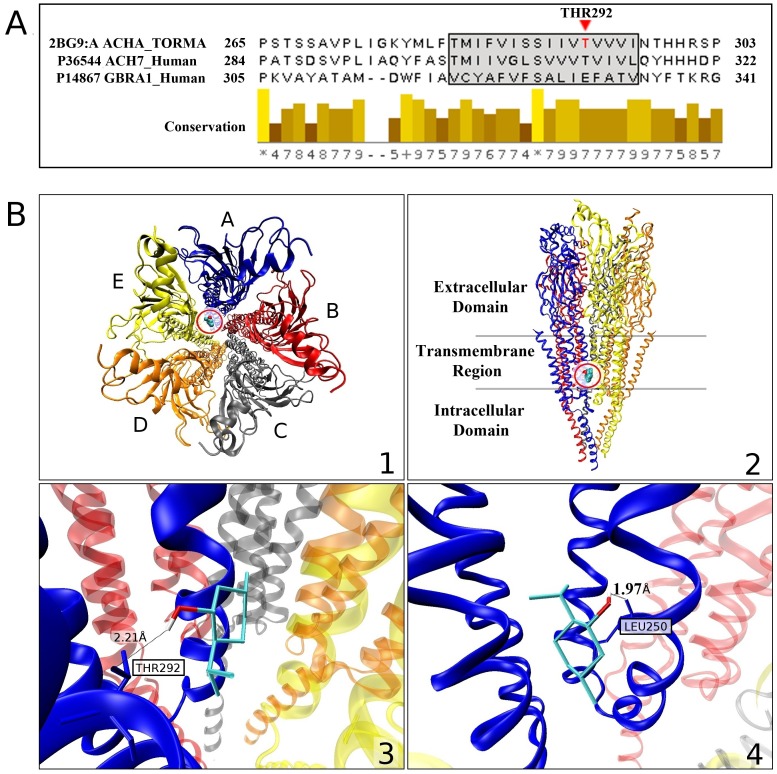
A multiple sequence alignment and conservation scores obtained with MUSCLE Vr.3.8.31 [30] between the human GABA_A_R-α_1_ subunit (UniProt AC: P14867), human α7-nACHR (UniProt AC:P36544 and the muscle nACh receptor subunit chain A ([24]; PDB ID: 2BG9) in (A). The fragment highlights, through the boxed and shaded region, key residue positions from the M3 segment of GABA_A_R-α_1_ evaluated by Williams and Akabas [29] for propofol binding. The docking simulation indicates binding of the menthol ligand on muscle nACh receptor residue THR292. This position is indicated by the red triangle and corresponds to the most frequent docking site. High sequence conservation about the binding site with muscle nACh receptor could indicate similar binding site characteristics for α7-nACh receptor. (**B**) Representative docked configuration for menthol (ZINC ID: 01482164) on the crystal structure of muscle nACh receptor ([24]; PDB ID: 2BG9). 1) Top-down view of nACh receptor with chain A colored in blue, B in red, C in gray, D in orange, and E in yellow. The binding site for the ligand is circled. As the α7-nACh receptor is a homopentamer, this conserved binding site could also be found on all five receptor subunits of the functional receptor. 2) Side-view of the circled binding site. 3) Lowest-energy (−6.15 kcal/mol) configuration, on four of the ten simulations resulting in lowest interaction energies, is shown for the L-menthol ligand. The h-bond that stabilizes the ligand onto the crystal structure is formed at residue THR292 of the muscle nACh receptor chain A (blue) at a distance of 2.21 Å. 4) The second most-frequent configuration for the ligand, corresponding to interaction energy of −5.98 kcal/mol, obtained on two of the top ten simulations. An h-bond with residue LEU250 of chain A (blue) at a distance of 1.97 Å stabilizes this docked configuration.

To survey the molecular properties of menthol interaction with the nACh receptor we utilized structural docking studies using the nACh muscle receptor; currently, the only complete nACh receptor available in the Protein Data Bank [23], and menthol. A protein sequence alignment underscores homology between the muscle nACh receptor and the α7-nACh receptor (Fig. 7A). A subset of residues, annotated by the red triangle (Fig. 7A), are found to constitute a possible binding site for menthol on the nACh receptor using this docking simulation approach.

An analysis of ligand placements with the lowest interaction energies suggests key residues of menthol binding within the crystal structure of the muscle nACh receptor. An illustration of a docked configuration for menthol and the muscle nACh receptor reveal an h-bond stabilizing menthol association with the nACh receptor (Fig. 7B panels 3 and 4). This h-bond involves residue THR292 of the muscle nACh receptor chain A at a distance of 2.21 Å. Four of the top ten (lowest-energy) docking configurations for menthol were found to involve this residue (Fig. 7B panel 3). Another placement of menthol, noticed on two of the ten lowest-energy configurations (corresponding to the second lowest interaction energies of −5.98 kcal/mol) involves LEU250 of the muscle nACh receptor chain A (Fig. 7B panel 4). In this case, menthol is found to form an h-bond at a distance of 1.97 Å. While this section of the sequence alignment is not visible in Fig. 7A, the α7-nACh receptor was found to also have a LEU residue at the corresponding position. These results suggest that residues THR292 and LEU250 of the nACh receptor, as based on the crystal structure of the muscle receptor, can play a key role in menthol binding. Because of the high sequence homology between the muscle and the α7-nACh receptor, at these sites, these findings are applicable to possible menthol interactions with the α7-nACh receptor. Moreover, it is interesting to point out that since the α7-nACh receptor is a homopentamer, each of the subunits appears to maintain a possible menthol binding site.

## Discussion

In this study, we provide novel evidence on an interaction between menthol and the α7- nACh receptor. Our study suggests that menthol inhibits α7-nACh receptors in a non-competitive manner thus likely not binding to the ACh site on the receptor. Studies in cultured neural cells that endogenously express the α7-nACh receptor evidence on the effect of menthol on α7-nACh receptor activity in neural cells suggesting that menthol targets nACh receptors within the brain. At this point of analysis however, we cannot conclude that menthol directly binds the nACh receptor. Based on structural modeling studies, a possible menthol binding appears to exist within the nACh receptor class thus presenting an important direction of interest in receptor mutagenesis studies.

In earlier studies, participation of G-protein coupled receptors such as kappa-opioid receptors [32] and the involvement of G-proteins in menthol [39] and nicotine [19] induced cellular responses have been reported. Our results indicate that the effect of menthol is not sensitive to pertussis toxin thus excluding the possible role of G-protein signaling in its cellular effect. Menthol has also been shown to increase intracellular Ca^2+^ levels and activate various Ca^2+^ sensitive kinases [2]. In *Xenopus* oocytes, activation of α_7_-nACh receptors, due to their high Ca^2+^ permeability, allows sufficient Ca^2+^ entry to activate endogenous Ca^2+^-dependent Cl^−^ channels [34]. In oocytes injected with BAPTA and recorded in a solution containing 2 mM Ba^2+^, menthol was found to inhibit α_7_-nACh receptor-mediated ion currents, suggesting that Ca^2+^-dependent Cl^−^ channels are not involved in the effect of menthol on the nACh receptor. In addition, because the reversal potential in solutions containing Ba^2+^ was not altered in the presence of menthol, the inhibitory effects of menthol appear to be not related to changes in the Ca^2+^ permeability of the α_7_-nACh receptor-channel. Furthermore, since Ca^2+^-activated Cl^−^ channels are highly sensitive to intracellular Ca^2+^ levels (for reviews, [35], [36]) alterations in intracellular Ca^2+^ levels would be reflected by changes in the holding current under voltage-clamp conditions. However, during our experiments, application of menthol, even at the high concentrations (300 µM) used in this study, did not cause alterations in the holding current, suggesting that menthol does not affect intracellular Ca^2+^ concentrations.

Open-channel blockade is a widely used model to describe the block of ligand-gated ion channels [40]. However, this model does not appear to account for our results based on two key observations: 1. Unlike open channel blockers, in which the agonist is required to allow the channel blocker to enter the channel after a conformational change, pre-application of menthol was found to augment its own inhibition of the α7-nACh receptor (Fig. 2), suggesting that menthol interacts with the closed state of the receptor; 2. inhibition by menthol appears to be not voltage sensitive, suggesting that the menthol-binding to the channel is not affected by the transmembrane electric field.

Menthol, in the concentration ranges used in this study, has been shown to act directly on the several ligand-gated ion channels including GABA-A ([38]; EC_50_ = 1.1 mM), glycine ([41]; 100–300 µM), and the α4β2 nACh receptor ([12]; IC_50_ = 111 µM). In addition, menthol appears to modulate a number of voltage-gated ion channels ([38]; IC_50_ = 297 µM for Na^+^ channels and IC_50_ of 125 µM Ca^2+^ channels in dorsal horn neurons). We find that menthol concentrations capable of producing an effect on the α_7_-nACh receptor in *Xenopus* oocytes are lower then the concentrations found to activate TRPM8 channels [42]. Menthol non-selectively also activates TRPV3 (EC_50_ 20 mM), inhibits mouse TRPA1 (IC_50_ = 68 µM) [43]. In our study, the concentration of menthol effective on human α7-nACh receptor ranged from 3 µM to 1 mM (IC_50_ = 32.6 µM). Similar concentrations of menthol were found effective on endogenous α7-nACh receptor in rat neuroendocrine cells. These concentrations approximate those used in human psychophysical studies and are considerably lower than those used in over-the-counter products (≈500 mM) [44], [45]. Menthol taken orally is effectively absorbed in gastro-intestinal mucosa and can easily reach the range of menthol concentrations used in this study suggesting that can act α7-nACh receptors within humans.

Based on electrophysiological studies, we find that only the efficacy, and not the potency, of ACh was inhibited by menthol. We propose that that menthol does not compete with ACh to the same binding site on the α7-nACh receptor. In agreement with this, our radioligand binding studies indicate that the specific binding characteristics of [^125^I] α-bungarotoxin, which shares the same binding site as ACh, are also not affected by menthol. Using computational modeling we find that menthol binds the nACh receptor at LEU and THR at sequence positions 250 and 292 respectively (Fig. 7). While modeling is based on the structure of the muscle nACh receptor, these menthol binding sites appear conserved in the human α7-nACh receptor subunit. Collectively, these findings indicate that menthol can act as an allosteric inhibitor of the α7-nACh receptor a property allowing it to modulate the receptor at various concentrations of ACh or nicotine. Interestingly, in the concentration range used in this study, menthol has been reported to inhibit the activity of acetylcholine esterase, [46], [47]. The inhibition of nicotine-induced [^3^H]NE release by menthol indicates that the actions of menthol observed in the expression systems and single cells also occur in neurons and may therefore contribute to neuronal circuitry and function.

Interaction between menthol and nACh receptors has been studied in several earlier investigations [9], [11], [12]. Nicotine, a major irritant contained in tobacco smoke [48], elicits burning or stinging pain sensation on oral or nasal mucosa [9]. Nicotine induced sensations are thought to involve activation of nACh receptors, including those composed of the α7 subunit, expressed in the sensory fibers innervating these tissues [49] and in bronchial and tracheal epithelia of the pulmonary tissue [50], [51]. Nicotine induced irritation and sensory perception is reduced by menthol [9]. Recently, menthol has been shown to act as counter-irritant against inhaled cigarette smoke [11] suggesting that nicotine-induced responses are reduced by menthol. In addition to sensory responses, one of the major physiological effects of nicotine is a decrease in body temperature due to cutaneous vasodilation, an action originating in brain probably mediated by hypothalamic nicotinic receptors [52]. Both chronic and acute menthol administrations diminish the effect of nicotine on body temperature [10].

It is interesting to consider that menthol, a common cigarette additive, has been associated with a greater tobacco dependence potential and lower success in cessation attempts [3], [7], [4]. A reduction in α7 nACh receptor function has been proposed to constitute a biological mechanism for increased motivation for cigarette smoking [53], [54]. Several earlier genetic studies demonstrate that reductions in α7-nACh receptor function result in significant elevations in motivation to self-administer nicotine [55], [56]. Similarly, antagonism of α7 nACh receptors in the anterior cingulate cortex was found to be sufficient to increase nicotine self-administration [54]. Based on these findings, it is likely that higher levels of nicotine addiction observed in mentholated cigarette users [57] involve antagonistic actions of menthol on α7 nACh receptors.

Menthol is known to act stereo-selectively in some, but not all, *in vivo* and *in vitro* assay systems (for reviews, 1, 2]. In an earlier study, Hall et al [41] showed that the effect of menthol on GABA_A_ currents were stereo-selective with (+)-menthol being more potent than (−)-menthol, while menthol modulation of glycine-receptors did not display stereo-specificity. In our study, we could not detect a stereo-selectivity of menthol actions on α7-nACh receptor (Fig. 1). Cyclohexane (100 µM), aromatic skeleton of menthol, displayed undetectable efficacy at inhibiting α7-nACh receptor. The substitution pattern on the cyclohexane skeleton and an aromatic hydroxyl group caused a significant increase in the potency of menthol and propofol in inhibiting α7-nACh receptor. The results observed for the parent compound cyclohexane and derivatives thereof may be useful in further understanding the molecular mechanisms involved in pharmacological effects of menthol as well as propofol. Other terpenes with close structural similarities to menthol, such as camphor [58] and borneol [59] have also been shown to inhibit the function of nACh receptors in a noncompetitive manner in chromaffin cells. Clearly, further structure-activity relationship studies are required in future investigations. These data add to a growing body of evidence [2] suggesting that in addition to TRPM8 receptors, α7-nACh receptors are pharmacologically targeted by menthol in cells.
